# Perceptions of HIV Seriousness, Risk, and Threat Among Online Samples of HIV-Negative Men Who Have Sex With Men in Seven Countries

**DOI:** 10.2196/publichealth.7546

**Published:** 2017-06-20

**Authors:** Anna N Chard, Nicholas Metheny, Rob Stephenson

**Affiliations:** ^1^ Department of Environmental Health Rollins School of Public Health Emory University Atlanta, GA United States; ^2^ Department of Health Behavior and Biological Sciences School of Nursing University of Michigan Ann Arbor, MI United States; ^3^ Center for Sexuality and Health Disparities School of Nursing University of Michigan Ann Arbor, MI United States

**Keywords:** sexual minorities, HIV, risk, perception

## Abstract

**Background:**

Rates of new HIV infections continue to increase worldwide among men who have sex with men (MSM). Despite effective prevention strategies such as condoms and pre-exposure prophylaxis (PrEP), low usage of both methods in many parts of the world hinder prevention efforts. An individual’s perceptions of the risk of acquiring HIV and the seriousness they afford to seroconversion are important drivers of behavioral risk-taking. Understanding the behavioral factors suppressing the uptake of HIV prevention services is a critical step in informing strategies to improve interventions to combat the ongoing HIV pandemic among MSM.

**Objective:**

The study aimed to examine cross-national perceptions of HIV/AIDS seriousness, risk, and threat and the association between these perceptions and sociodemographic characteristics, relationships, and high-risk sexual behaviors among MSM.

**Methods:**

Participants in Australia, Brazil, Canada, Thailand, South Africa, the United Kingdom, and the United States were recruited for a self-administered survey via Facebook (N=1908). Respondents were asked to rate their perceived seriousness from 1 (not at all serious) to 5 (very serious) of contracting HIV, their perceived risk from 1 (no risk) to 10 (very high risk) of contracting HIV based on their current behavior, and their perception of the threat of HIV—measured as their confidence in being able to stay HIV-negative throughout their lifetimes—on a scale from 1 (will not have HIV by the end of his lifetime) to 5 (will have HIV by the end of his lifetime). Covariates included sociodemographic factors, sexual behavior, HIV testing, drug use, and relationship status. Three ordered logistic regression models, one for each outcome variable, were fit for each country.

**Results:**

Contracting HIV was perceived as serious (mean=4.1-4.6), but perceptions of HIV risk (mean=2.7-3.8) and threat of HIV (mean=1.7-2.2) were relatively low across countries. Older age was associated with significantly lower perceived seriousness of acquiring HIV in five countries (Australia: odds ratio, OR 0.97, 95% CI 0.94-0.99; Brazil: OR 0.95, 95% CI 0.91-0.98; Canada: OR 0.96, 95% CI 0.93-0.98; South Africa: OR 0.96, 95% CI 0.94-0.98; United Kingdom: OR 0.95, 95% CI 0.92-0.98). Being in a male-male sexual relationship was associated with significantly lower perceived risk of HIV in four countries (Australia: OR 0.47, 95% CI 0.30-0.75; Canada: OR: 0.54, 95% CI 0.35-0.86; United Kingdom: OR 0.38, 95% CI 0.24-0.60; United States: OR 0.5, 95% CI 0.31-0.82). Drug use in the previous year was associated with greater threat of contracting HIV in two countries (Canada: OR 1.81, 95% CI 1.13-2.91; United Kingdom: OR 1.7, 95% CI 1.06-2.74).

**Conclusions:**

Few measures of behavioral or sexual risk-taking were significantly associated with perceived HIV seriousness, risk, or threat across countries. Overall, low levels of reported risk were identified, and results illustrate important gaps in the understanding of risk among MSM across societies that could be addressed through culturally-tailored prevention messaging.

## Introduction

Although new HIV infections are stable or decreasing among other risk groups, incident infections continue to increase among men who have sex with men (MSM) in the United States [1], Western Europe, and Australia [2]. In low and middle-income countries, MSM have 19-fold greater odds of testing HIV sero-positive compared with the general adult male population [3]. The quality and coverage of HIV prevention services varies dramatically across the world. In many low- and middle-income countries, lack of MSM-centric HIV prevention programs, low rates of HIV knowledge, and low rates of condom use exist among MSM [4,5]. In these countries, low prevention method uptake is driven largely by societal stigma, homophobia, and constrained resources for MSM outreach [4]. In many resource-rich countries, the inverse is often a cause for low prevention uptake among MSM. Prevention fatigue, or safer sex fatigue, refers to the attitude that HIV prevention messaging and programs have become tiresome because of frequent and targeted interventions and social marketing campaigns regarding safe-sex behaviors since the beginning of the pandemic [6,7]. In both resource-rich and resource-poor settings, the advent of treatment as prevention (TasP) and pre-exposure prophylaxis (PrEP) provide a promising biomedical method for preventing incident HIV infections. Yet, large numbers of MSM in both resource-rich and resource-poor settings are unaware of their sero-status [8,9], and PrEP usage rates remain low, with studies reporting use at less than 3% among MSM in samples across resource-rich and resource-poor settings [10,11]. However, with a significant scale-up in the use of these prevention methods, incident rates could fall dramatically [12,13], with one model showing a 70% reduction in new HIV cases when 90% of those who are HIV-positive are virally suppressed and PrEP usage is at 65% among those who are eligible [14]. Understanding the behavioral factors suppressing the uptake of HIV prevention services is a critical step in informing strategies to scale up promising biomedical interventions.

Several theories focused on understanding HIV-related risk behaviors such as the Health Belief Model and the Theory of Planned Behavior, which assert that perceptions of HIV susceptibility severity drive HIV-negative individuals’ motivations to use HIV prevention methods [15]. Therefore, in order to increase HIV prevention uptake, accurate assessment of HIV risk is important. Although recent work has found that many MSM underestimate their risk of contracting HIV [16-21], there is a scarcity of research addressing what drives perceptions of HIV seriousness, risk, and threat among HIV-negative MSM. This study fills gaps in this knowledge in two key ways. First, the authors found no previous studies in which MSM quantify their perceptions of HIV seriousness, risk, or threat, providing comparable, standardized data across multiple countries. Second, this is the first study to examine cross-national perceptions of HIV and AIDS seriousness, risk, and threat, and the association between these perceptions and sociodemographic characteristics, relationships, and high-risk sexual behaviors among MSM. Results from this study may be influential in informing HIV prevention uptake strategies for HIV-negative MSM internationally.

## Methods

### Data

Participants were recruited online for a self-administered survey. Banner ads were placed on Facebook and displayed to men who indicated an interest in men only on their profiles and reported living in Australia, Brazil, Canada, South Africa, Thailand, the United Kingdom, or the United States. These countries were selected because of their large populations of self-identified gay and bisexual men on Facebook and to allow comparisons across socioeconomic and cultural contexts. Clicking on the advertisement led potential participants to information regarding the survey. After obtaining electronic informed consent, participants were presented with the eligibility screener that affirmed that the participants were born male, were over 18 years old, and self-reported anal or oral sex with a man in the previous year. Except for the Brazilian and Thai surveys, which were administered in Portuguese and Thai, respectively, all surveys were administered in English. Ads were displayed until 500 men in each country were recruited (range= 5 days (United States) to 14 days (Thailand)). The study was approved by the Institutional Review Board (IRB determination IRB00047677).

A total of 11,850 people across the seven sample countries clicked on the advertisement and were exposed to the eligibility screener. Of these, 58.01% (6874/11,850) began the eligibility screener; 13.09% (1551/11,850) did not meet eligibility criteria and were disqualified from completion. Among the 5323 eligible participants, 54.27% (2889/5323) men completed the survey. Of the men who completed the survey, 6.44% (186/2889) reported being HIV-positive and 27.52% (795/2889) had never been tested for HIV. Thus, they did not meet the criteria for this analysis of being known to be HIV-negative. A final convenience sample of 1908 participants who met all eligibility criteria provided data for all covariates of interest and was included in the analysis.

The anonymous survey took approximately 30 minutes to complete and collected information on participants’ sociodemographic characteristics (including age; race, ethnicity, or ancestry; and level of education). Age of sexual debut with a man was self-reported as the age at which the respondent first had sex (type of sex not defined) with a man; drug use was assessed by asking whether respondents had used any nonprescription drugs in the previous 12 months. Relationship status was determined by asking respondents whether they were currently in a sexual relationship and whether that relationship was with a man. Participants self-reported whether they had ever had an HIV test; respondents answering affirmatively were asked the date of their last HIV test. To evaluate the percentage of all condomless anal intercourse (CAI) engaged in by respondents in the previous year and the percentage of lifetime anal sex partners encountered in the previous year, respondents were asked the numbers of male anal sex partners and condomless male anal sex partners they had had intercourse with in the past 12 months and in their lifetimes.

The outcome variables were developed specifically for this survey from a review of the literature that highlighted three distinct domains of risk perception: perception of the seriousness of HIV, perception of the risk of acquiring HIV based on current behavior, and perception of the ability to remain HIV negative. The authors then developed simple scale items designed to address each of these domains. To measure perceptions of HIV seriousness, respondents were asked to rate their perception of the seriousness of contracting HIV (“How serious for you would it be if you contracted HIV?”) on a scale from 1 (not at all serious) to 5 (very serious), their perception of the risk of contracting HIV based on their current behavior (“How would you rate your risk for contracting HIV based on your current behavior?”) on a scale from 1 (no risk) to 10 (very high risk), and their perception of the threat of HIV (“How confident are you that you can stay HIV-negative in your lifetime?”) on a scale from 1 (will not have HIV by the end of his lifetime) to 5 (will have HIV by the end of his lifetime). These three questions provide three outcome variables measuring (1) the perceived seriousness of contracting HIV, (2) the perceived risk of contracting HIV, and (3) the perceived threat of HIV. Respondents were not compensated for completing the survey.

### Analysis

Data were cleaned and analyzed using Stata version 12 (StataCorp, 2012). As respondents were asked to rate their perceived risk, seriousness, and threat of contracting HIV in the future, only participants reporting having ever been tested for HIV and having had a known HIV-negative status were included in the analysis. The key sociodemographic covariates included age; education, which was dichotomized as receiving a secondary school education or less (≤12 years) or receiving higher education (>12 years); race, categorized as nonminority race or ethnicity (Australia: European, Brazil: white, Canada: European, South Africa: black, Thailand: Thai, United Kingdom: white, and United States: white) or minority race or ethnicity (Australia: other; Brazil: mixed, brown, or Other; Canada: Other; South Africa: white or other; Thailand: other; United Kingdom: black or other; United States: black or other), and drug use in the previous 12 months. Currently being in a sexual relationship was dichotomized as being in a relationship with a man versus being single.

Covariates measuring sexual behaviors included age of sexual debut with a man; percentage of condomless versus all anal intercourse partners encountered in the previous year, which was calculated by dividing respondents’ reported number of CAIpartners in the previous year by the reported number of all anal intercourse partners in the previous year; and percent of lifetime anal sex partners encountered in the previous year, which was calculated by dividing the reported number of anal sex partners in the previous year by the total number of lifetime anal sex partners. Time since respondents’ last HIV test was calculated in years by subtracting the month and year of survey completion from the reported month and year of respondents’ last HIV test. Given the ordinal outcomes of perceived HIV seriousness, risk, and threat, the analysis employed ordered logistic regression models. In order to make cross-country comparisons of the outcome variables, separate models were fitted for each of the three outcomes in each of the seven countries. This required separate datasets for each country, fitting three models per dataset.

## Results

### Demographic Factors

Demographic characteristics of the sample, respondents’ sexual behaviors, time since respondents’ last HIV test, and perceived seriousness, risk, and threat of HIV seroconversion are summarized in Table 1. The mean age of respondents ranged from 25.8 (Brazil) to 33.9 (Canada). The majority of respondents reported having 12 years or more of education, belonging to a majority race (except in South Africa), currently being in a sexual relationship with a man, and no drug use in the previous 12 months.

Across all the countries, between 50.2%-63.8% of respondents’ anal sex partners in the previous year were condomless, and less than 1/3 of respondents’ anal intercourse partners had been encountered in the previous year. Time since respondents’ most recent HIV test ranged from 1.2 (South Africa) to 2.3 (Thailand and the United Kingdom) years.

Country-specific mean perceived seriousness of contracting HIV ranged from 4.1 (SD=0.1) in Thailand to 4.7 (SD=0.9) in the United States (see Table 2), mean perceived risk of contracting HIV ranged from 2.7 (SD=2.0) in Canada to 3.8 (SD=0.2) in Thailand (see Table 3), and mean perceived threat of HIV ranged from 1.7 (SD=0.9) in Canada and the United Kingdom to 2.2 (SD=0.1) in Thailand (see Table 4).

Respondent age was negatively associated with perceived HIV seriousness in all countries except Thailand and the United States. Age was negatively associated with perceived risk in the United States. There was a significant, positive association between education and perceived HIV seriousness, risk, and threat in two countries. Compared to respondents with less than or equal to 12 years of education, respondents with more than 12 years of education had significantly greater odds of perceiving a higher HIV seriousness in the United Kingdom and a higher HIV risk and threat in Canada. Moreover, in Canada, respondents belonging to minority races had significantly lower odds of perceiving a higher HIV seriousness than respondents belonging to nonminority races. South African respondents belonging to a minority race had significantly lower odds of perceiving a higher HIV risk and HIV threat than those belonging to a nonminority race.

**Table 1:** Respondent characteristics and mean minority stress scale index scores of 1891 internet-recruited men who have sex with men in seven countries.

Variable		Australia (n=274), μ (%)	Brazil (n=294), μ (%)	Canada (n=274), μ (%)	South Africa (n=386), μ (%)	Thailand (n=146), μ (%)	United Kingdom (n=280), μ (%)	United States (n=254), μ (%)
Age in years		30.4 (11.5)	25.8 (7.7)	33.9 (12.6)	33.2 (10.3)	32.3 (7.8)	30.6 (11.2)	30.9 (13.4)
**Education**								
	≤12 years	103 (37.6)	97 (33.0)	64 (23.4)	118 (30.6)	22 (15.1)	62 (22.1)	71 (28.0)
	>12 years	171 (62.4)	197 (67.0)	210 (76.6)	268 (69.4)	124 (84.9)	218 (77.9)	183 (72.1)
**Race/Ethnicity^a^**								
	Nonminority	168 (61.3)	189 (64.3)	219 (79.9)	56 (14.5)	138 (94.5)	266 (95.0)	207 (81.5)
	Minority	106 (38.7)	105 (35.7)	55 (20.1)	330 (85.5)	8 (5.5)	14 (5.0)	47 (18.5)
**Drug use in previous 12 months**								
	No	153 (55.8)	203 (69.1)	145 (52.9)	233 (60.4)	112 (76.7)	179 (63.9)	162 (63.8)
	Yes	121 (44.2)	91 (30.9)	129 (47.1)	153 (39.6)	34 (23.3)	101 (36.1)	92 (36.2)
**Relationship Status**								
	Single	130 (47.5)	150 (51.0)	107 (39.1)	165 (42.8)	60 (41.1)	122 (43.6)	118 (46.5)
	In a relationship	144 (52.6)	144 (49.0)	167 (60.9)	221 (57.3)	86 (58.9)	158 (56.4)	136 (53.5)
Age of sexual debut with a man		17.5 (4.7)	15.2 (3.9)	17.4 (5.0)	17.1 (4.6)	17.9 (4.4)	17.5 (4.6)	17.1 (4.8)
Percentage of unprotected versus all anal intercourse partners in previous year		54.7 (42.8)	50.2 (41.2)	54.4 (43.9)	63.8 (42.5)	54.0 (43.7)	55.9 (43.2)	61.1 (43.7)
Percentage of lifetime anal sex partners encountered in previous year		26.5 (29.5)	30.1 (29.3)	25.6 (29.3)	26.3 (25.7)	35.0 (33.6)	28.1 (29.3)	31.8 (34.0)
Years since most recent HIV test		1.4 (2.5)	1.4 (1.9)	1.7 (2.6)	1.2 (1.9)	2.3 (2.7)	2.3 (4.1)	1.5 (2.7)
Perceived seriousness of contracting HIV (1-10)		4.6 (0.9)	4.6 (0.9)	4.6 (1.0)	4.3 (1.1)	4.1 (0.1)	4.6 (0.9)	4.7 (0.9)
Perceived risk of contracting HIV (1-5)		2.9 (2.1)	3.2 (2.4)	2.7 (2.1)	3.0 (2.4)	3.8 (0.2)	2.8 (2.1)	3.0 (2.3)
Perceived inability to stay HIV-negative throughout life (1-10)		1.8 (1.0)	2.1 (1.1)	1.7 (0.9)	1.8 (1.0)	2.2 (0.1)	1.7 (0.9)	1.8 (1.0)

^a^Non-minority race/ethnicity was categorized as follows: Australia: European, Brazil: white, Canada: European, South Africa: black, Thailand: Thai, United Kingdom: white, United States: white. Minority race/ethnicity was categorized as follows: Australia: Other; Brazil: Mixed, brown, and Other; Canada: Other; South Africa: white and Other; Thailand: Other; United Kingdom: black and Other; United States: black and Other.

**Table 2:** Adjusted Odds Ratios and 95% CIs between perceived HIV seriousness and respondent characteristics.

Variable	Australia (n=274), aOR (95% CI)	Brazil (n=294), aOR (95% CI)	Canada (n=274), aOR (95% CI)	South Africa (n=386), aOR (95% CI)	Thailand (n=146), aOR (95% CI)	United Kingdom (n=280), aOR (95% CI)	United States (n=254), aOR (95% CI)
Age in years	0.97 (0.94-0.99)^a^	0.95 (0.91-0.98)^a^	0.96 (0.93-0.98)^a^	0.96 (0.94-0.98)^a^	0.97 (0.93-1.01)	0.95 (0.92-0.98)^a^	0.98 (0.95-1.01)
≤12 years of education (ref: >12 years)	1.21 (0.65-2.28)	0.95 (0.53-1.71)	0.64 (0.29-1.43)	0.85 (0.52-1.39)	1.84 (0.71-4.77)	1.94 (1.02-3.71)^a^	0.94 (0.45-1.92)
Minority race (ref: nonminority race)	1.11 (0.59-2.09)	1.15 (0.65-2.02)	0.4 (0.19-0.82)^a^	1.3 (0.67-2.51)	0.4 (0.09-1.72)	1.3 (0.34-4.97)	0.79 (0.34-1.83)
Drug use in previous 12 months (ref: no drug use)	0.88 (0.47-1.66)	0.84 (0.48-1.45)	1.44 (0.76-2.72)	1.07 (0.68-1.69)	0.9 (0.41-1.97)	0.49 (0.28-0.87)^a^	0.86 (0.44-1.67)
In a relationship (ref: single)	1.17 (0.61-2.24)	0.8 (0.47-1.34)	1.03 (0.52-2.02)	0.64 (0.4-1.03)	0.48 (0.24-0.98)^a^	0.84 (0.47-1.51)	1.26 (0.62-2.56)
Age of sexual debut with a man	1.08 (1.01-1.16)^a^	1.02 (0.95-1.08)	1.09 (1.02, 1.17^a^	1.02 (0.97-1.07)	1.01 (0.93-1.1)	1.04 (0.98-1.11)	0.98 (0.92-1.04)
Percentage of unprotected versus all anal intercourse partners in previous year	0.87 (0.4-1.88)	0.92 (0.49-1.75)	0.93 (0.44-1.96)	1.76 (1.02-3.05)^a^	1.65 (0.74-3.7)	1.5 (0.76-2.94)	0.63 (0.28-1.45)
Percentage of lifetime anal sex partners encountered in previous year	1.8 (0.45-7.25)	0.76 (0.32-1.8)	4.38 (0.85-22.56)	1.33 (0.5-3.58)	1.73 (0.57-5.28)	1.62 (0.47-5.56)	1.3 (0.41-4.13)
Years since most recent HIV test	1 (0.9-1.12)	1.08 (0.94- 1.24)	1.04 (0.91-1.18)	0.96 (0.87-1.07)	0.97 (0.87-1.09)	1.08 (1.01-1.15)^a^	0.93 (0.83-1.05)

^a^Statistical significance, alpha=.05.

**Table 3:** Adjusted Odds Ratios and 95% CIs between perceived HIV risk and respondent characteristics.

Variable	Australia (n=274), aOR (95% CI)	Brazil (n=294), aOR (95% CI)	Canada (n=274), aOR (95% CI)	South Africa (n=386), aOR (95% CI)	Thailand (n=146), aOR (95% CI)	United Kingdom (n=280), aOR (95% CI)	United States (n=254), aOR (95% CI)
Age in years	0.98 (0.96-1)	1 (0.97-1.03)	1 (0.98, 1.02)	1.01 (0.99-1.03)	0.97 (0.94-1.01)	1 (0.97-1.03)	0.97 (0.95-0.99)^a^
≤12 years of education (ref: >12 years)	0.75 (0.48-1.18)	1.17 (0.74-1.84)	1.83 (1.06, 3.16)^a^	1.3 (0.87-1.96)	0.77 (0.34-1.73)	1.31 (0.74-2.33)	1.55 (0.92-2.62)
Minority race (ref: nonminority race)	1.24 (0.78-1.95)	1.18 (0.76-1.81)	1.37 (0.79, 2.39)	0.53 (0.3-0.91)^a^	0.49 (0.14-1.71)	0.69 (0.26-1.85)	0.75 (0.4-1.37)
Drug use in previous 12 months (ref: no drug use)	1.36 (0.86-2.14)	1.39 (0.89-2.16)	2.6 (1.67, 4.05)^a^	1.78 (1.22-2.58)^a^	1.77 (0.88-3.55)	2.23 (1.41-3.52)^a^	1.85 (1.16-2.95)^a^
In a relationship (ref: single)	0.47 (0.3-0.75)^a^	0.73 (0.48-1.11)	0.54 (0.35, 0.86)^a^	0.75 (0.51-1.1)	1.31 (0.7-2.42)	0.38 (0.24-0.6)^a^	0.5 (0.31-0.82)^a^
Age of sexual debut with a man	0.95 (0.9-0.99)^a^	0.94 (0.9-1)^a^	1 (0.96, 1.05)	0.99 (0.95-1.03)	1.06 (0.98-1.15)	1 (0.95-1.05)	1 (0.95- 1.05)
Percentage of unprotected versus all anal intercourse partners in previous year	0.65 (0.37-1.12)	0.5 (0.3-0.84)^a^	0.9 (0.53, 1.53)	0.69 (0.44-1.1)	0.98 (0.48-2.01)	0.67 (0.4-1.12)	1.07 (0.6-1.89)
Percentage of lifetime anal sex partners encountered in previous year	1.05 (0.45-2.43)	1.33 (0.67-2.66)	1.28 (0.56, 2.92)	1.99 (0.91-4.34)	0.86 (0.35-2.09)	1.65 (0.71-3.8)	1.04 (0.48-2.27)
Years since most recent HIV test	0.93 (0.83-1.04)	1.06 (0.95-1.19)	0.95 (0.87, 1.05)	0.94 (0.86-1.04)	1.18 (1.05-1.31)^a^	0.92 (0.87-0.98)^a^	1 (0.9-1.1)

^a^Statistical significance at alpha=.05.

### Behavioral Factors

There was a significant negative association between drug use and perceived HIV seriousness in the United Kingdom. Conversely, there was a significant positive association between drug use and perceived HIV risk in Canada, South Africa, the United Kingdom, and the United States and between drug use and threat of HIV in Canada and the United Kingdom.

Reporting a current sexual relationship with a man was negatively associated with perceived HIV seriousness (Thailand), risk (Australia, Canada, the United Kingdom, and the United States), and threat (Australia) in several countries. As compared with those not in a male-male relationship, respondents reporting currently being in a sexual relationship had significantly lower odds of perceiving a higher HIV seriousness in Thailand; significantly lower odds of perceiving a higher risk of HIV in Australia, Brazil, Canada, the United Kingdom, and the United States; and significantly lower odds of perceiving a higher threat of HIV in Australia.

Age of sexual debut with a man was positively associated with perceived seriousness of HIV in two countries and negatively associated with perceived risk of HIV in two countries. As age of sexual debut with a man increased, the odds of perceiving a higher seriousness of HIV increased significantly in Australia and Canada, while the odds of perceiving a higher HIV risk significantly decreased in Australia and Brazil. As the percentage of condomless anal intercourse partners in the previous year increased, the odds of perceiving a higher HIV risk significantly decreased in Brazil and the odds of perceiving a higher HIV threat significantly decreased in Brazil and South Africa. The percent of lifetime anal sex partners encountered in the previous year was associated with only one outcome (HIV threat) in one country. In the United States, the odds of perceiving a higher HIV threat significantly decreased as the percentage of lifetime anal sex partners encountered in the previous year increased. Time since last HIV test was positively associated with perceived HIV seriousness in the United Kingdom. The association between time since last HIV test and perceived HIV risk yielded mixed results. As time since respondents’ last HIV test increased, odds of perceiving a higher HIV risk increased significantly among participants in Thailand, but decreased significantly among participants in the United Kingdom.

**Table 4:** Adjusted Odds Ratios and 95% CIs between perceived HIV threat and respondent characteristics.

Variable	Australia (n=274), aOR (95% CI)	Brazil (n=294), aOR (95% CI)	Canada (n=274), aOR (95% CI)	South Africa (n=386), aOR (95% CI)	Thailand (n=146), aOR (95% CI)	United Kingdom (n=280), aOR (95% CI)	United States (n=254), aOR (95% CI)
Age in years	0.99 (0.96-1.01)	1.02 (0.98-1.05)	1 (0.98-1.03)	1.01 (0.99-1.03)	0.99 (0.95-1.03)	1.02 (0.99-1.04)	0.99 (0.97-1.01)
≤12 years of education (ref: >12 years)	0.87 (0.54-1.42)	1.29 (0.81-2.05)	2.27 (1.23-4.19)^a^	1.37 (0.89-2.1)	0.46 (0.19-1.11)	1.06 (0.58-1.94)	1.31 (0.75-2.27)
Minority race (ref: nonminority race)	1.2 (0.74-1.94)	0.74 (0.47-1.16)	0.83 (0.45-1.54)	0.53 (0.3-0.95)^a^	2.17 (0.59-7.88)	1.06 (0.37-3.01)	1.55 (0.82-2.92)
Drug use in previous 12 months (ref: no drug use)	1.42 (0.87-2.31)	1.33 (0.84-2.12)	1.81 (1.13-2.91)^a^	1.4 (0.94-2.08)	1.22 (0.6-2.51)	1.7 (1.06-2.74)^a^	0.9 (0.55-1.48)
In a relationship (ref: single)	0.55 (0.34-0.89)^a^	0.75 (0.49-1.16)	0.84 (0.51-1.38)	0.94 (0.63-1.4)	1.15 (0.6-2.2)	0.7 (0.44-1.11)	0.78 (0.46-1.32)
Age of sexual debut with a man	0.98 (0.93-1.04)	0.98 (0.93-1.03)	0.98 (0.93-1.03)	1 (0.96-1.04)	1.05 (0.97-1.13)	1 (0.95-1.05)	1 (0.95-1.06)
Percentage of unprotected versus all anal intercourse partners in previous year	0.74 (0.41-1.34)	0.54 (0.32-0.93)^a^	0.89 (0.5-1.58)	0.52 (0.32-0.84)^a^	1.4 (0.67-2.94)	0.81 (0.47-1.41)	0.55 (0.3-1.02)
Percentage of lifetime anal sex partners encountered in previous year	0.65 (0.26-1.61)	1.21 (0.59-2.49)	1.11 (0.43-2.87)	1.2 (0.52-2.76)	1.17 (0.45-2.99)	0.96 (0.38-2.42)	0.36 (0.15-0.87)^a^
Years since most recent HIV test	1.03 (0.92-1.15)	1 (0.89-1.13)	0.98 (0.89-1.08)	0.97 (0.88-1.07)	1.08 (0.97-1.21)	0.94 (0.88-1.01)	0.92 (0.82-1.03)

^a^Statistical significance at alpha=.05.

## Discussion

### Principal Findings

The results of this study point to the potential influence of drug use, relationships, and age on individual reporting of perceived HIV seriousness, risk of HIV seroconversion, and lifetime threat of HIV seroconversion among MSM across culturally and economically diverse settings. While some cross-national trends were found, the variations across countries point to the importance of cultural context in understanding the drivers of perceived risk of HIV.

Respondents reporting currently being in a sexual relationship with a man perceived a lower risk of contracting HIV compared to respondents not in a relationship. This may be the result of messaging to MSM regarding HIV risk. Since the beginning of the epidemic, HIV prevention messaging has focused on the risks associated with having multiple concurrent sexual partners. However, recent evidence suggests that between one-third [22] to two-thirds [23] of incident HIV infections among MSM result from a main partner, a pattern driven by more frequent anal intercourse and more infrequent condom use between main partners relative to casual partners [23,24]. Recent research also shows that US MSM with main partners are less likely to test regularly for HIV than single MSM [25]. Central to understanding the differences in HIV risk behaviors between single and partnered men is the idea that partnered men perceive being in a relationship as protective, regardless of their outside sexual behavior or their knowledge of their partners’ serostatus [25]. The findings of this study reinforce this idea: partnered men in a range of social and cultural contexts perceived themselves to be at less risk of HIV acquisition. The models did not control for the presence of a sexual agreement in the relationship: it may be that the partners are monogamous and have knowledge of each other’s serostatus and are thus correct in their estimation of a lowered risk of HIV. Despite this, the results suggest the need for ongoing research attention to understand how partnered men perceived their HIV risks and the extent to which this varies by context, as well as greater programmatic attention to the unique needs of partnered men. For example, Couples HIV Counseling and Testing (CHCT) allows partnered men to examine HIV prevention as a dyad [26-28].

Older respondents reported a significantly lower perceived seriousness (five countries) and risk (US) of contracting HIV or AIDS than younger respondents. These results may reflect some degree of treatment optimism; largely as a result of highly active anti-retroviral treatment (HAART), HIV-positive MSM are living longer than they were twenty years ago. Although the sample is of HIV-negative men, these men may have observed the changes in the impact of HIV on those living with it, —and the transformation of HIV into manageable chronic condition. This may allow them to perceive the threat of HIV as less serious now than in the past. The prevalence of HIV and AIDS among older MSM is higher than among young MSM in many places [29,30], although incidence is increasing among younger MSM. Several studies have identified having older MSM sex partners to be an independent risk factor for HIV transmission [30,31] because older MSM have had more lifetime sexual partners and sexual encounters, and thus more opportunities for HIV seroconversion. Our findings suggest that decreased perceptions of the seriousness and risk of HIV acquisition might be an issue for older MSM in the countries sampled and point to the need for HIV prevention messaging and services to be adapted to specifically reach older MSM who may be underestimating their HIV risk and need for prevention.

Few measures of recent sexual behavior were significantly associated with perceived HIV seriousness, risk, or threat across multiple countries. An older age of sexual debut with a man was associated with a higher perceived HIV seriousness (Australia and Canada) and lower perceived HIV risk (Australia and Brazil). It is possible that a higher perceived seriousness of HIV actually delayed participants’ sexual debut. An earlier age of sexual debut has been associated with engaging in sexual risk behaviours and positive HIV status [32,33]. We have observed the inverse; perhaps a later age of sexual debut may in turn be associated with more conservative sexual behaviors or fewer sexual partners, leading to participants’ perception of a lower risk of contracting HIV. Additionally, despite a higher perceived seriousness of HIV among men with a greater percentage of CAI partners in the previous year in South Africa, perceived risk (Australia and Brazil) and threat (Brazil and South Africa) were lower among participants in some countries. The discordance between sexual risk taking and perceptions of HIV seriousness, risk, and threat may reflect some degree of cognitive dissonance or HIV fatalism among these participants [34,35]. The knowledge of treatment as prevention (TasP) may be another reason for a reduced risk perception among men with a higher percentage of CAI, with the assumption that an undetectable viral load equates to an inability to transmit HIV. While being on PrEP or engaging in CAI with a partner on PrEP may also reduce risk perception, the survey was administered before the widespread availability of PrEP in these countries. Therefore, its use does not likely factor into decreased HIV risk and threat perceptions. In contrast, in multiple countries, participants reporting drug use in the previous year perceived their risk and threat of HIV to be higher than non-drug using respondents. It is possible that MSM using drugs identify the increased risks of HIV acquisition associated with drug use (through needle sharing or drug-linked sexual risk-taking). It is also possible that drug use is a maladaptive response to social pressure and discrimination, which acts to increase both the use of drugs and the perception of increased risk of HIV acquisition. In some contexts, such as environments where homosexuality is highly stigmatized, drug use may be a strategy to mitigate stress associated with internalized homonegativity and to normalize same-sex thoughts, feelings, and behaviours and connect with others in the gay community [36-38].

### Limitations

There are limitations to the present study, most of which result from the Web-based convenience sampling design. In all countries, the survey was advertised only to men who were registered users of Facebook and had a profile indicating an interest in men, leading to selection bias towards men who may be more public about their sexual identity or behavior. A recent study reported that Web-based recruitment and venue-based recruitment yields similar samples of MSM in the United States [39]; however, whether this holds true in international settings is yet to be established. An additional limitation is that a significant proportion of those who clicked on the banner ads did not complete the survey; we do not have data on their characteristics to establish the extent of this selectivity bias. The use of a one-year recall period may not be ideal for assessing the number of sex partners and could lead to biased responses. Another limitation is the lack of a country-specific context to this study. While we focus on the broad, international patterns of HIV seriousness, risk, and threat in this publication, future research at the country level should include a discussion of the cultural, political, and historical factors that may affect these perceptions among MSM. Despite these limitations, however, this study demonstrated the usefulness of a Web-based survey tool in reaching MSM across countries and collecting standardized data across economically and culturally-diverse settings with large samples of men who have sex with men.

### Conclusions

Perceived risks of HIV infection were generally low across MSM in this international sample. Across countries, older MSM and those in sexual relationships perceived themselves to have lower risk and threat of HIV, while drug using MSM perceived their HIV risk as higher. Few measures of sexual behavior were associated with perceptions of HIV seriousness, risk, or threat. Our results point to the need for more nuanced research into the constructs of risk perception among MSM. Currently, little is known about how risk perception varies with age, stage of life, or the availability of new biomedical preventative options such as PrEP. Among MSM internationally, varying sociopolitical climates and the relative strength of structural minority stressors such as heteronormative social pressure and homophobic discrimination may also have important effects. A more cogent understanding of the constructs associated with HIV risk perception may lead to more tailored and effective HIV prevention interventions for MSM. The results also suggest that, while existing HIV prevention messages continue to focus on the HIV risks of condomless sex, such messages may need to be packaged differently for men in relationships and for older men. Additionally, novel strategies to help MSM understand the true risks of unsafe sex and to surmount the effects of behavioral discordance are still needed.

## Abbreviations

AIDS: acquired immune deficiency syndrome
CAI: condomless anal intercourse
CHCT: couples HIV counseling and testing
HAART: highly active anti-retroviral treatment
HIV: human immunodeficiency virus
MSM: men who have sex with men
aOR: adjusted Odds Ratio
